# Experimental Performances of Titanium Redox Electrodes as the Substitutes for the Ruthenium–Iridium Coated Electrodes Used in the Reverse Electrodialysis Cells for Hydrogen Production

**DOI:** 10.3390/membranes16010026

**Published:** 2026-01-03

**Authors:** Zhaozhe Han, Xi Wu, Lin Xu, Ping He

**Affiliations:** 1 Key Laboratory of Ocean Energy Utilization and Energy Conservation of Ministry of Education, School of Energy and Power Engineering, Dalian University of Technology, Dalian 116024, China; 2 School of Civil Engineering, Liaodong University, Dandong 118001, China; linlin19872008@126.com

**Keywords:** reverse electrodialysis, hydrogen production, electrode, hydrogen evolution reaction, membrane

## Abstract

Reverse electrodialysis (RED) enables the efficient conversion of the chemical potential difference between seawater and freshwater into electricity while simultaneously facilitating hydrogen production for integrated energy utilization. Nevertheless, the widespread deployment of RED remains constrained by the reliance on ruthenium–iridium-coated electrodes, which are expensive and resource-limited. This study proposes the adoption of titanium-based redox electrodes as a replacement for traditional precious metal electrodes and employs a novel spike structure to accelerate hydrogen bubble detachment. The electrochemical performance of titanium electrodes in an RED hydrogen production system was systematically evaluated experimentally. The influences of several parameters on the RED system performance were systematically examined under these operating conditions, including the ruthenium–iridium catalytic layer, operating temperature (15 to 45 °C), electrode rinse solution (ERS) concentration (0.1 to 0.7 M), and flow rate (50 to 130 mL·min^−1^). Experimental results demonstrate that optimized titanium redox electrodes maintain high electrocatalytic activity while significantly reducing system costs. Under optimal conditions, the hydrogen yield of the Ti redox electrode reached 89.7% of that achieved with the mesh titanium plate coated oxide iridium and oxide ruthenium as electrodes, while the electrode cost was reduced by more than 60%. This is also one of the cost-cutting solutions adopted by RED for its development.

## 1. Introduction

As countries around the world pursue rapid industrialization, traditional fossil energy has become increasingly unable to meet the growing energy demands. This has led to an increasing number of environmental problems and phenomena such as global warming. Salinity gradient energy (SGE), existing between seawater and freshwater, is regarded as the one of the clean energy sources. Recent studies have demonstrated that reverse electrodialysis (RED) is a promising technology capable of both recovering energy from the natural salinity gradients and treating concentrated brine [1,2]. The RED equipment can convert the SGE into electrical power directly, and even store it as hydrogen, which has gained significant attention as an alternative green energy source [3,4].

The RED system operates by passing two salt solutions of different concentrations through alternately arranged cation and anion-exchange membranes. This membrane unit is composed of the cation-exchange membranes (CEMs), anion-exchange membranes (AEMs), and spacers arranged alternately. The concentrated solution (HC) and dilute solution (LC) flow inside of the respective flow channels of the membrane unit. Under the driving force of the concentration gradient, anions and cations on the HC side migrate in opposite directions towards the adjacent LC flow channels via the AEM and CEM, respectively. As a consequence, the ionic current is generated on the membrane sides, thereby forming an electric potential difference. Oxidation-reduction reactions occur in the electrode chamber to achieve hydrogen production in the cathode chamber and oxygen production in the anode chamber [5]. This technology not only recovers natural SGE between the seawater and the river but also addresses environmental issues related to artificial concentrated brine disposal, potentially making desalination more sustainable by reducing its ecological footprint [6,7].

The historical origins of RED technology can be traced back to 1954 when Pattle first proposed using salinity gradients for power generation, achieving 3.1 V electromotive force and 0.2 W/m^2^ power density using 47 pairs of acid–base membranes [8]. In 2016, Tufa et al. [9] first demonstrated the feasibility of utilizing SGE for indirect hydrogen production. They integrated an RED unit with an alkaline polymer-electrolyte water electrolyzer and employed NaCl solutions ranging from 0.1 M to 5 M to simulate various water sources. Under their experimental conditions, the maximum hydrogen yield reached 44 cm^3^·h^−1^·cm^−2^, and the system operated stably. The study also revealed that a high concentration difference (5 M–0.1 M) generated higher open circuit voltages (OCVs) and power densities. This work laid a foundation for the practical application of RED-based hydrogen production [9].

In the development of RED-based hydrogen production, reducing the overpotentials for the hydrogen evolution reaction (HER) and oxygen evolution reaction (OER) is crucial. Studies [10,11] have indicated that replacing the traditional neutral solutions in the anode and cathode chambers with acidic or alkaline solutions can effectively lower the cell voltage and overpotentials for these reactions. In 2020, Moya analyzed the uphill transport in improved reverse electrodialysis by removal of divalent cations in the dilute solution based on the Nernst–Planck transport equations [12]. Two years later, Solberg et al. [13] investigated the thermally driven RED heat engine (REDHE) concept that employs a precipitation-based regeneration unit. Comparing evaporation and precipitation regeneration schemes, the study showed that the evaporation option demands 40 times less waste heat and delivers threefold higher hydrogen production. With commercial evaporation technology, the system reaches 2% efficiency and yields H_2_ 0.38 g·m^−2^·h^−1^. The water-transport and salt-diffusion coefficients of the membranes were identified as key parameters: raising the salt-diffusion coefficient from 10^−12^ to 10^−11^ drops the efficiency from 2% to 0.6%, underscoring the critical role of membrane performance. Researchers compared several thermolytic salt solutions, such as NH_4_HCO_3_ and KNO_3_, for the REDHE system. It was highlighted that the membrane water transport coefficient (t_e_w) and salt diffusion coefficient critically affect the system’s hydrogen production efficiency. As reported, REDHE efficiencies range between 0.6% and 24%, depending on membrane and solution properties, but specific quantitative relationships (e.g., changes in single-cell potential with t_e_w) were further elaborated in later modeling studies [14]. In 2024, Pellegrino et al. [3] developed and tested a 25-cell-pair RED–electrolysis coupled system that simultaneously concentrated salinity gradients between seawater and river water to produce electricity and hydrogen. A techno-economic assessment was also performed. Under optimal current conditions, the system achieved a hydrogen production rate of 3.1 mL cm^−2^ h^−1^ (≈0.45 m^3^ H_2_ m^−2^ d^−1^) with a Faradaic efficiency of 78%. Furthermore, it was estimated that treating 1 m^3^ of concentrated brine could yield about 0.45 m^3^ of hydrogen and 0.25 kWh of electricity as by-products.

Hatzell et al. [15] compared the two energy recovery methods of hydrogen and electricity in the RED system and found that when hydrogen was extracted from the electrode and collected, the system’s energy recovery per unit volume could reach 118 W·h·m^−3^, which was 1.5 times that of pure power generation (78 W·h·m^−3^). At the same time, they pointed out that the non-precious metal catalyst molybdenum boride (MoB) performed similarly to Pt in the HER and had the potential for low-cost substitution, proposing that direct hydrogen production was a more valuable energy utilization path for the RED system. Simões et al. [16] conducted experiments and modeling studies on the impact of electrode segmentation in RED on power and energy efficiency. Electrode segmentation can optimize local current density, increasing the net energy efficiency from 17% to 25%, and enhancing power density by 39%. This significantly reduces operating and equipment costs. The conclusion drawn is that electrode segmentation can improve energy efficiency without sacrificing power and is suitable for large-scale applications.

One of the key challenges in optimizing the RED system for hydrogen production is the influence of the electrode pair in the two electrode chambers on the overall system efficiency. While the precious metal catalytic layers do contribute to hydrogen production, their high cost presents a major obstacle to the industrial RED applications [17,18]. It is necessary to enhance the hydrogen production efficiency of the RED system by using cost-effective electrodes, which is achieved by improving their structural design and optimizing the operating parameters. Additionally, the RED system performance is influenced by several factors, such as the driven salinity gradient, solution ionic compositions, pH, etc. [19,20]. For example, under the higher pH conditions in the concentrated brine, the RED system’s power output can be improved to some extent, whereas the extreme pH values may reduce the performance of the membranes. Precisely balancing these variables is crucial for maximizing energy recovery and hydrogen production [11]. Given these challenges, integrating RED with other technologies like water electrolysis shows potential for further improving the performance and scalability [21,22]. Multistage reverse electrodialysis stacks are capable of recovering more energy from salinity gradients; moreover, the use of advanced electrode material is beneficial in reducing the dependence on precious metals such as ruthenium and iridium.

Based on the above research, the performance of a reverse electrodialysis system can be greatly enhanced essentially. This study mainly aims to make the low-cost titanium redox electrodes catch up with the high-cost catalytic electrodes in terms of energy efficiency by using the newly developed titanium redox electrodes and optimizing the electrode reaction condition. By combining the relatively matchable operating parameters and using the electrode pair with a unique structure, the economic efficiency and comprehensive competitiveness of hydrogen and electricity co-production in the RED system is improved.

## 2. Materials and Methods

### 2.1. Experimental Chemicals and Equipment

In the performance experiments where titanium redox electrodes were used to replace the titanium mesh electrode coated oxide iridium and oxide ruthenium (Ru-Ir-coated Ti mesh electrode) for hydrogen production via an RED system, the working solutions primarily consisted of NaCl solutions. The concentration of the dilute solution (LC) was 0.02 mol·L^−1^, while that of the concentrated solution (HC) was 4.50 mol·L^−1^. The electrode solution used in the RED stack was NaOH, with a concentration of 0.7 mol·L^−1^. Both NaCl and NaOH are of analytical purity (AR) grade and are manufactured by Tianjin Damao Chemical Reagent Ltd., Tianjin, China. All solvents used in this experiment were laboratory-prepared deionized water, and the solutes were dissolved in the solvent using a magnetic stirrer. The working solutions are pumped into the solution compartments using the peristaltic pumps. The new type of electrode used in the experiment was provided by the HuangJiangyuming 3D Modeling Design Department in Dongguan City, China. Its titanium content is as high as 99%. All the other electrodes are sourced from Suzhou Shurutai Industrial Technology Co., Ltd. in SuZhou City, China.

In this study, all the electrodes will experience a certain degree of oxidation. All titanium-containing electrodes will form a TiO_2_ oxide layer on their surface, and this oxidation process has a dual impact on the electrodes. The presence of the oxide layer will reduce the conductivity of the system, but it has self-repairing properties in air or solution, which also provides certain protection and prevents internal corrosion. When the current density is very low, the corrosion effect can be negligible. The lifespan of the electrodes is approximately several months. To ensure the accuracy of the experiments, all electrodes should be replaced after 100 h of use.

The equipment and instruments used in the experimental process are listed in Table 1. The fundamental principle of hydrogen and electricity cogeneration in the RED system has been discussed earlier. During the performance tests, an electrochemical workstation was used to measure the electrical output of the system, while a gas–liquid separator was employed to measure the hydrogen production efficiency. The connection setup is as follows: the electrochemical workstation is connected to an external current amplifier, which is in turn connected to a computer. The current amplifier expands the current range, facilitating the testing of the RED system’s power generation performance. A three-electrode electrochemical workstation was used in the laboratory, where one circuit consists of the working electrode connected to the cathode of the RED stack, and the other circuit consists of the auxiliary and reference electrodes connected to the anode of the RED stack. The cathode flow channel is connected to the H_2_ collector of the gas–liquid separator, while the anode flow channel is connected to the O_2_ collector.

### 2.2. Design of Experimental System

**Figure 1:**
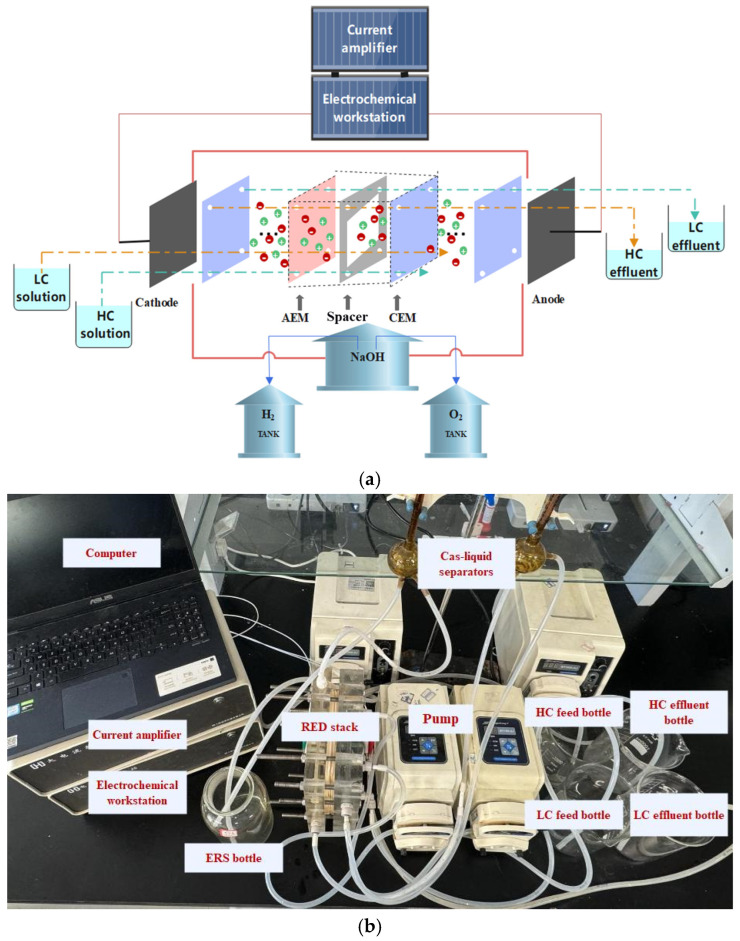
RED hydrogen production experimental system: (**a**) schematic diagram; (**b**) photograph.

Figure 1a illustrates the schematic diagram of the RED hydrogen–electricity cogeneration experimental system driven by the salinity gradient energy of HC and LC, in which different electrodes can be replaced. Figure 1b shows a photograph of the experimental setup. Figure 1 also depicts the arrangement of the CEMs, AEMs, and the mesh spacers in the RED device. The mesh spacers were custom-made in the laboratory and composed of polyethylene terephthalate (PET), the technical details of which can be seen from Table 2. A total of 60 mesh spacers were alternately placed between the 30 pairs of CEMs and AEMs. One more CEM was applied to seal the outermost layers of the membrane stack on both sides near the electrode chambers. It is because the NaCl working solution contains Cl^−^ ions, which could be oxidized into toxic Cl_2_ gas if they enter the chambers. The membranes used were Fuji Film Type 10, and the detailed parameters of the membranes are listed in Table 3. The directional ionic transport across the membranes generates a potential difference. The accumulated potential difference from the multiple membrane unit drives redox reactions at the electrode–solution interfaces, achieving hydrogen and electricity cogeneration within the RED stack.

In this experimental condition, the default setting is a room temperature of 25 °C degrees Celsius, an ERS concentration of 0.1 M, and an ERS flow rate of 90 mL·min^−1^. The RED system is supported by a pair of 20 cm × 25 cm acrylic plates (namely the end plates) with electrodes embedded at both ends. Between the end plates and the membranes lies a 10 cm × 10 cm electrode chamber at each side. In this experiment, the identical electrodes were used for either the anode or cathode. To enhance the system’s sealing and prevent leakage of the working solution near the electrode chamber due to pressure differences during operation, the leak-proof plates were added at the junction between the membranes and the electrode chamber. This design also protects the membranes from potential damage. It can also prevent the influence of Cl^−1^ and other ions on the reaction.

### 2.3. RED Evaluation Methods

The RED system undergoes redox reactions, with the overall reaction as follows:

(1) $$2H_{2}O\to 2H_{2}+O_{2}$$

The reduction reaction occurring at the cathode is as follows:

(2) $$4H_{2}O+4e^{-}\to 2H_{2}+4{OH}^{-}$$

The oxidation reaction occurring at the anode is as follows:

(3) $$4{OH}^{-}\to O_{2}+2H_{2}O+4e^{-}$$

The redox reactions occur under alkaline conditions, following the mechanisms described in hydrogen evolution reaction studies. HER can proceed through the following three main steps: the Volmer step, the Tafel step, and the Heyrovsky step. The corresponding reactions are as follows.

The Volmer step involves the adsorption and dissociation of water molecules on the electrode surface, generating adsorbed H* and hydroxide ions OH^−^:

(4) $$H_{2}O+e^{-}\to H_{ads}+{OH}^{-}$$

The Tafel step is one pathway for the hydrogen evolution reaction, involving the combination of two adsorbed hydrogen atoms H_ads_ to form a hydrogen gas molecule H_2_. The specific reaction is as follows:

(5) $$H_{ads}+H_{ads}\to H_{2}$$

The Heyrovsky step is another pathway for the hydrogen evolution reaction, involving the reaction of one adsorbed hydrogen atom H_ads_ with a water molecule to form a H_2_ and an OH^−^. The specific reaction is as follows:

(6) $$H_{ads}+H_{2}O+e^{-}\to H_{2}+{OH}^{-}$$

The formula for the *P*_d_ of a RED system is as follows:

(7) $$P_{d}=P/(N\cdot A_{IEMs})$$

In the formula, *P_d_* is the power density (W·m^−2^), *P* represents the power, *N* is the number of membrane pairs, and *A*_IEMs_ is the effective area of the membrane per unit (m^2^).

The open circuit voltage (OCV) of the RED system can be expressed by the Nernst equation, which is the sum of the membrane potentials of all membrane pairs:

(8) $$E_{OCV}=(\alpha_{CEM}+\alpha_{AEM})\frac{NRT}{ZF}ln\frac{\gamma_{HC}C_{HC}}{\gamma_{LC}C_{LC}}$$

In the equation, *α*_*CEM*_ and *α*_*AEM*_ are the selectivity coefficients for the CEM and AEM; *R* is the gas constant (8.314 J∙mol^−1^∙K^−1^); T is the thermodynamic temperature (K); *z* is the ion valence; *F* is the Faraday constant (96485 C·mol^−1^); *γ* is the activity coefficient of ions in the working solution; and C is the molar concentration of the solution (mol·L^−1^).

The output voltage (U) of the RED stack can be expressed as

(9) $$U=E_{OCV}-IR_{in}-E_{el}$$

In the equation, *I* represents the current; *R_in_* is the internal resistance of the membrane stack (Ω); *E_el_* is the voltage required for the electrochemical reactions at the electrode–ERS interface, including the reaction equilibrium potential, the overpotentials, and the voltage drop caused by the Ohmic resistance in the electrode chamber:

(10) $$E_{el}=E_{a}-E_{c}+|\eta_{a}|+|\eta_{c}|+Ir_{el}$$

In the equation, *E_a_* and *E_c_* represents the reaction equilibrium potential, which is the difference in the equilibrium potentials between the anode and the cathode, $r_{el}$ is the Ohmic resistance caused by the migration of electrolyte ions and the bubbles on the electrodes within the electrode chamber. |*η*_a_| and |*η*_c_| are the overpotentials for the OER and the HER, respectively, calculated using the Tafel equation:

(11) |η|=a+blog i

In the equation, *i* is the current density, *a* is a constant related to electrode performance, which also depends on temperature and solution conditions, and *b* is the Tafel slope.

Based on Faraday’s law and the ideal gas law, the theoretical hydrogen production of the RED stack can be expressed as ($m_{thH2}$, mol·m^−2^·h^−1^):

(12) $$m_{thH2}=\frac{It}{2F}\frac{RT}{p}$$

In the equation, *p* is the atmospheric pressure; and *t* is the duration of the test (s).

The Faradaic efficiency of the hydrogen evolution reaction ($\eta_{H2}$) is defined as the ratio of the experimental hydrogen production (m_H2_) to the theoretical hydrogen production (m_thH2_):

(13) $$\eta_{H2}=\frac{m_{H2}}{m_{thH2}}$$

## 3. Results and Discussions

### 3.1. Performance of the Mesh Titanium Plate Coated Oxide Iridium and Oxide Ruthenium as Electrodes

**Figure 2:**
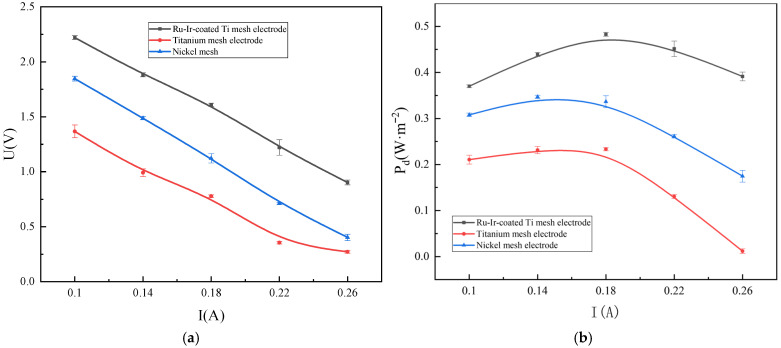
The variation of output voltage (**a**) and power with current density (**b**) in three different electrode hydrogen-electricity cogeneration systems.

Figure 2 shows the variation trends of the output voltage and power density of the RED system under three different electrode conditions. As the current density gradually increases, the RED system’s output voltage continuously decreases. With the increase in current, the electrochemical polarization on the electrodes intensifies, leading to an increase in the electrode reaction overpotential and a consequent drop in output voltage. Meanwhile, the ion transport across the membranes is accompanied by concentration polarization and variations in ion concentration along the flow path, which result in an increase in non-ohmic internal resistance [23].

**Figure 3:**
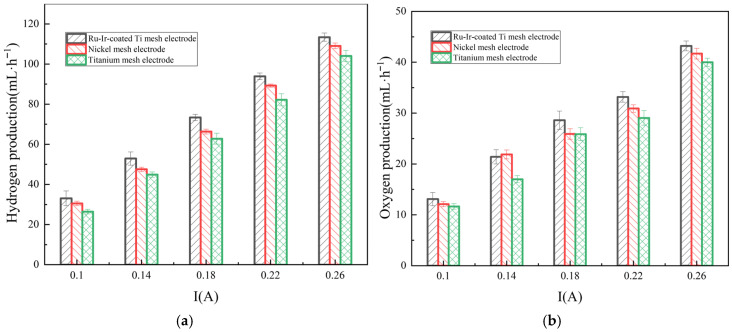
The variation of hydrogen (**a**) and oxygen (**b**) production with current in three different electrode hydrogen-electricity cogeneration systems.

As shown in Figure 3a, the Ru-Ir-coated Ti mesh electrode demonstrates markedly higher hydrogen production than Ti and Ni mesh electrodes. Increasing the current from 0.1 A to 0.26 A enhances hydrogen generation in the RED system, indicating a strong dependence on current density. At the current condition of 0.26 A, the average hydrogen production rates are 99.24, 107.26, and 113.4 mL·min^−1^ for Ti-, Ni-, and Ti-coated Ru-Ir mesh electrodes, respectively, which corresponds to the efficiency improvements of 14.27% and 5.73% for the Ti-coated Ru-Ir electrode relative to the Ti and Ni meshes. The superior performance arises from the RuO_2_-IrO_2_ layer anchored on the Ti substrate, which provides high metal dispersion and abundant active sites at the electrode–ERS interface, facilitating adsorption of hydrogen intermediates and enhancing catalytic activity. In alkaline media, HER proceeds via Volmer, Heyrovsky, and Tafel steps, as shown in Section 2.3. In the Volmer step, the RuO_2_-IrO_2_ surface enables H_2_O dissociation and H* adsorption; excessively strong or weak adsorption reduces efficiency. The synergistic effect of RuO_2_ and IrO_2_ optimizes H adsorption energy, balancing adsorption, and desorption. During the Heyrovsky and Tafel steps, the layer’s stability and conductivity promote electron transfer and H* desorption, improving the overall HER performance. These findings align with the recent studies on Ti-doped RuO_2_ structure and catalytic behavior [24]. These results demonstrate the considerable potential of Ti-basis electrodes for application in the field of RED technology.

Figure 3b illustrates the variation in oxygen production in the RED system using three types of electrodes. The Ru-Ir-coated Ti mesh electrode exhibits significantly higher oxygen evolution than the Ti mesh electrode. During the OER process, the RuO_2_-IrO_2_ coating acts as a catalyst, enhancing the activity of hydroxide ions and thereby promoting the oxygen evolution. OER is characterized by sluggish anodic kinetics and high overpotentials. The Ru-Ir-coated Ti mesh electrode serves as an efficient catalyst to reduce the energy consumption. In the formation of the OH* process, the metal sites on the RuO_2_-IrO_2_ coating surface provide a strong adsorption, facilitating OH^−^ activation. The subsequent deprotonation of OH* involves overcoming a high energy barrier, where RuO_2_ contributes via its acidic sites to promote H^+^ release and formation of O*. During O–O bond formation, the strong oxidative capability of RuO_2_ accelerates the process. Finally, the generated O_2_ molecules desorb from the catalyst surface into the ERS; excessive adsorption can block active sites, but the RuO_2_-IrO_2_ catalytic layer optimizes desorption, thereby accelerating O_2_ release. These results substantiate the feasibility of transforming waste metals into high-performance electrodes [25], offering significant support for the economic viability of RED technology. During the reaction process, the ratio of H_2_ to O_2_ produced is not 2:1. This is because the anode may undergo an imperfect reaction. Under low overpotential conditions, water oxidation may follow a two-electron pathway, generating excess H_2_O_2_ instead of the four-electron formation of O_2_. Additionally, the electrode material will also undergo a certain degree of oxidation during the reaction, which may absorb some oxygen.

The HER and OER efficiencies of Ti and Ni mesh electrodes are significantly lower than that of the Ru-Ir-coated Ti mesh electrode. During the Volmer step of HER, Ti electrodes lack the sufficient surface active sites, making it difficult to overcome the high energy barrier for H_2_O dissociation, and the associated overpotentials are relatively high, which hinders the reaction. In the case of OER, Ti exhibits weak adsorption of OH^−^, limiting the formation of intermediates, while the extremely high overpotential further impedes the reaction.

### 3.2. Performance of the Titanium Redox Electrodes with Specially Designed Structure

**Figure 4:**
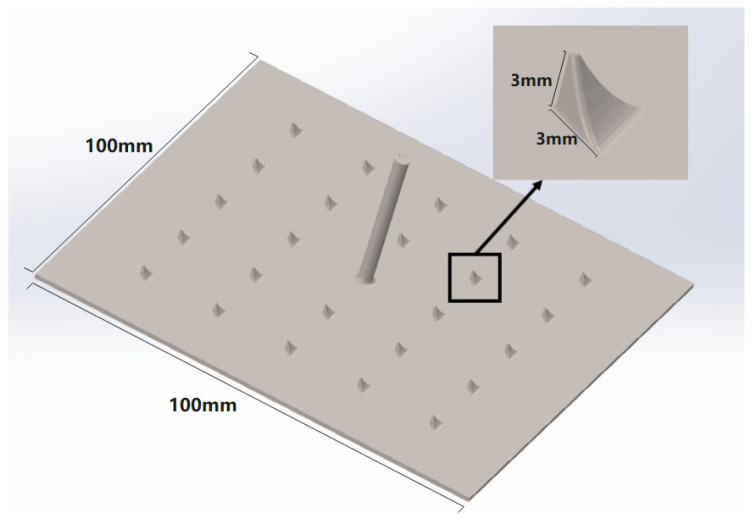
Simulated diagram of the novel protruded electrode.

This section investigates the feasibility of a newly designed protruded electrode, as Figure 4 shows, developed for enhancing hydrogen evolution. The electrode was fabricated using 3D printing equipment, and featuring regular triangular pyramidal protrusions. A total of 24 protrusions were distributed on the Ti substrate plate, with two faces perpendicular to the base plate, each of which has a height and base length of 3 mm, and the third face forming a quarter-circle arc. The specific surface area of this new type of electrode is greater than that of the traditional noble metal mesh electrode. However, its hydrogen production efficiency is still relatively low. This section aims to modify its operating conditions so that its hydrogen production efficiency can catch up with that of the precious metal electrodes.

#### 3.2.1. Under Varying HER Temperature Conditions

**Figure 5:**
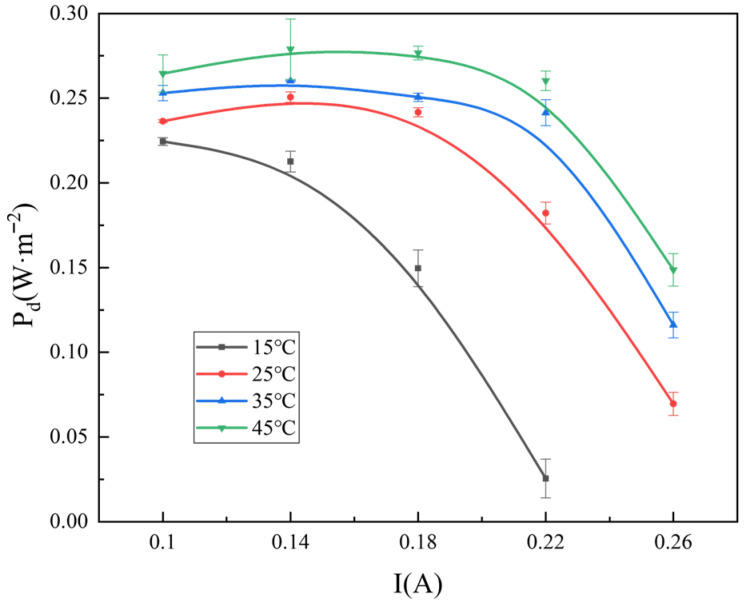
Variations in power density with respect to current in the hydrogen-electric cogeneration system using ERS at different temperatures.

The current density of the RED system increases moderately with the rising temperature, as shown in Figure 5. When the current is 0.14 A, the power density at 45 °C is 31.2% higher than that at the 15 °C condition, accompanied by a 14.1% increase in hydrogen production. The temperature condition affects the reaction kinetics, ionic conductivity, gas solubility, and overpotentials. An increase in temperature generally enhances the ionic conductivity of the ERS, accelerating OH^−^ migration in alkaline solutions, reducing resistance, and thereby decreasing ohmic losses. These results are in good agreement with previous detailed studies on the influence of temperature on hydrogen production in RED systems [4]. When the reaction rate is slow, an additional voltage is required to overcome the activation overpotential.

Increasing the ERS temperature accelerates the reduction of H^+^ to H_2_ and the oxidation of OH^−^ to O_2_ at the electrode surfaces, thereby lowering the activation overpotentials. According to the Arrhenius equation, $k\propto e^{-E_{a}/(RT)}$, the activation energy of the electrode reactions can be partially overcome by raising the solution temperature. Additionally, the higher temperature reduces the viscosity of the ERS, thereby increasing the diffusion coefficient, enhancing ion transport and convective mass transfer, and decreasing the concentration gradient at the electrode surface. These benefits are helpful for reducing the concentration polarization overpotential.

**Figure 6:**
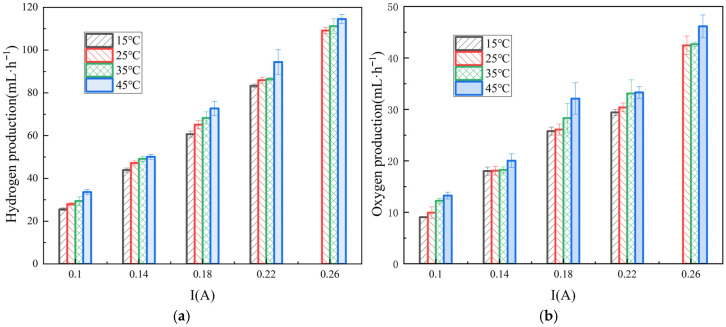
Variations in hydrogen (**a**) and oxygen production (**b**) with respect to current in the hydrogen-electric cogeneration system using ERS at different temperatures.

According to Henry’s law, the solubility of H_2_ and O_2_ in the solution decreases with increasing temperature, promoting the bubble nucleation and rapid detachment from the electrode surface, thereby reducing bubble coverage and local resistance. As Figure 4 and Figure 6 show, the spiked electrode was employed to facilitate bubble detachment from the electrode surface. When the gas bubbles adhere to the electrodes, they block the active sites, limiting reactions to uncovered regions, which reduces the effective surface area and consequently lowers the hydrogen evolution rate. This local reduction in active area forces an increase in current density, elevating overpotential and hindering ion diffusion from the ERS to the electrode surface. The spiked structure alters the surface morphology, promoting bubble nucleation at the spike tips. By reducing the contact area between the bubbles and electrode, the adhesion force is decreased. It allows bubbles to detach at the smaller volumes under the fluid shear, thereby minimizing surface coverage and maintaining a larger effective reaction area. The diffusion layer near the electrode surface is the primary resistance region for mass transfer. Bubble coverage thickens this layer and slows reactant diffusion. Upon detachment behaviors, bubbles induce the local fluid flow, thereby disrupting the static diffusion layer and reducing its thickness. A thinner diffusion layer corresponds to a higher mass transfer coefficient, thus enhancing reactant supply. During the hydrogen evolution in the RED system, H_2_ bubbles do not form at fixed positions on the electrode; however, the presence of spikes provides regular nucleation sites, thus promoting the uniform bubble distribution and preventing ‘dead zones’ caused by the random nucleation and localized over-coverage. Regarding the bubble detachment dynamics, the spiked structure modifies the detachment angle compared to planar surfaces, adjusting electrode wettability and promoting sliding detachment rather than the direct lift-off, which significantly shortens the bubble departure time.

Increasing the ERS temperature significantly enhances both the hydrogen and oxygen evolution efficiencies in the RED process. This improvement arises since the redox reactions in the electrode compartments must overcome the activation energy barriers. At the elevated temperature conditions, the charge transfer rates are accelerated, and the additional activation overpotential required for electrode reactions are reduced. Simultaneously, the ionic conductivity of the ERS increases with temperature, ion migration rates are enhanced, and viscosity decreases, facilitating the transport of H^+^ and OH^−^ to the electrode surface. Consequently, when substituting non-noble metal electrodes for conventional titanium mesh electrodes coated with oxide iridium and oxide ruthenium, the external operating conditions can be adjusted to improve the performance. From an industrial perspective, utilizing the waste heat (<100 °C) as an additional strategy to drive the RED system [26] would offer significant integrated economic and environmental advantages.

#### 3.2.2. Under Varying ERS Concentration Conditions

**Figure 7:**
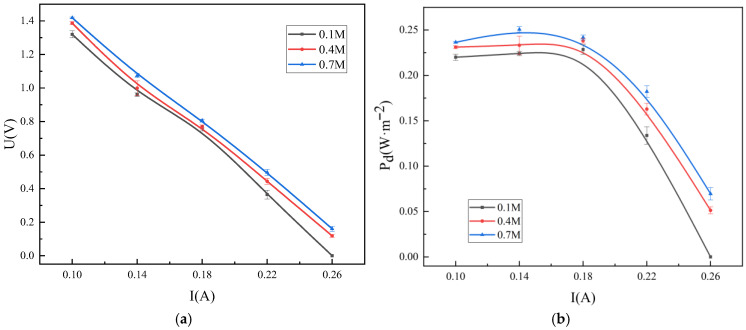
Variations in output voltage (**a**) and power density (**b**) with respect to current in the hydrogen-electric cogeneration system using ERS at different concentrations.

This section investigates the influence of ERS concentration on the RED system performance. Figure 7 shows the output voltage and power density of the RED system at different ERS concentrations. With an increase in the ERS concentration from 0.1 M to 0.7 M, there is a 5.8% average increase in output voltage and power density. It indicates that the effect of the ERS concentration on these parameters is relatively modest under the tested conditions. From a conductivity perspective, the higher the ERS concentration, the bigger the ionic conductivity. Increasing the Na^+^ and OH^−^ concentrations can improve the charge transport; however, when the consumption rate of OH^−^ at the electrode surface exceeds the replenishment rate from the bulk solution, the concentration polarization occurs. The limiting current density is increased directly as a result of elevating the OH^−^ concentration, which allows operation at higher current densities without significant concentration gradients. Additionally, the higher ERS concentration strengthens the diffusion driving force, thinning the diffusion layer and improving mass transfer efficiency [27]. ERS concentration gradients are a primary driving force in RED systems, directly affecting open-circuit voltage and power density. Optimizing the concentration gradient can thus significantly enhance energy conversion efficiency.

**Figure 8:**
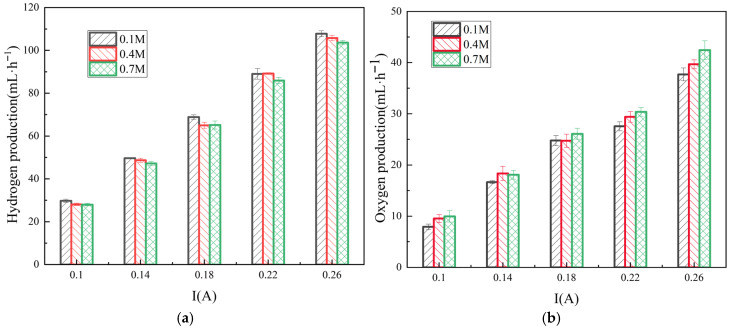
Variations in hydrogen (**a**) and oxygen production (**b**) with respect to current in the hydrogen-electric cogeneration system using ERS at different concentrations.

Figure 8a illustrates the effect of ERS concentration on the hydrogen evolution efficiency in the RED system. As the ERS concentration increases from 0.1 M to 0.7 M, hydrogen evolution efficiency decreases, which is consistent with the findings of Ernst and Hamann. As they report [28], in alkaline ERSs, the HER performance reaches a maximum at a hydroxide ion activity of approximately 0.1 mol·L^−1^. Other studies have also confirmed that in alkaline media, alkali metal cations influence the dissociation of O-H bonds in water molecules [29]. Under the alkaline conditions of pH values within 11 to 13, the cation concentration at the electrode interface increases with the solution pH value. These cations interact with the dissociated water molecules and stabilize the adsorbed hydrogen and intermediates generated during hydrolysis, thereby indirectly enhancing HER activity. However, the further increases in pH (above 13) can lead to cation saturation even exceeding an affordable threshold, thus causing competition with the adsorbed hydrogen atoms from the active sites on the electrode and reducing the HER activity. When the ERS concentration surpasses a critical value, this can enhance the ion–ion interactions but increase the solution viscosity, which in turn raises the ionic migration resistance. As a consequence, it increases the ERS’s ohmic resistance and requires a higher energy input to maintain the reaction. Figure 8b shows that increasing the ERS concentration slightly improves the OER. In the anodic OER process, O_2_ generation depends on the OH^−^ oxidation. A higher ERS concentration accelerates the charge transfer, enhancing the driving force for the reaction.

#### 3.2.3. Under Varying ERS Flow Conditions

**Figure 9:**
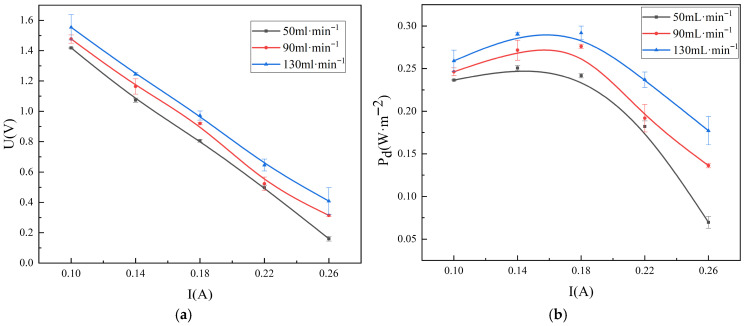
Variations in output voltage (**a**) and power density (**b**) with respect to current in the hydrogen-electric cogeneration system using ERS at different flow rates.

Figure 9 illustrate the effect of ERS flow rate over the protruded electrode on the RED system’s output voltage and power density. As the flow rate increases from 50 mL·min^−1^ to 130 mL·min^−1^, both the output voltage and power density of the RED system increase, this is consistent with the results of reference [30]. At a current of 0.18 A, the power density at ERS flow rate of 130 mL·min^−1^ are 18.6% higher than those at 50 mL·min^−1^. In the electrode chamber, a faster flow rate implies a shorter residence time of the ERS [31]. During reactions in the electrode compartments, the ions near the electrode surface (e.g., H^+^ or OH^−^) are rapidly consumed, which will create the local depletion and concentration polarization. As a result of which, an additional voltage is required to overcome the overpotential. The increase of the ERS flow rate serves to rapidly renew the reactants at the electrode surface, suppressing concentration gradients and consequently lowering the concentration polarization overpotential. This capability permits operation at either a lower voltage for the same current density, or at higher current densities without incurring voltage spikes. In the specialized structure of electrode used here, the higher flow rates increase the fluid shear, which accelerates bubble detachment. This reduces surface bubble coverage and thereby lowers ohmic resistance, enabling operation at higher current densities for increased power density. Furthermore, enhanced convective mass transfer at elevated flow rates thins the diffusion layer and improves ionic transport efficiency, further mitigating polarization. Consequently, by optimizing ERS flow rates, non-noble Ti-electrodes can achieve improved functionality in the RED systems.

**Figure 10:**
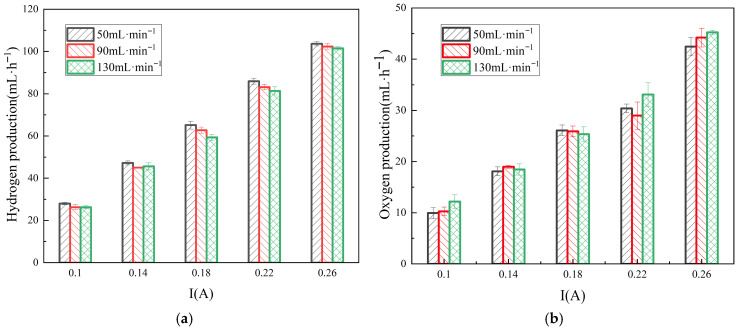
Variations in hydrogen (**a**) and oxygen (**b**) production with respect to current in the hydrogen-electric cogeneration system using ERS at different flow rates.

As Figure 10 shows, although increasing the ERS flow rate in the RED hydrogen cogeneration system enhances power generation performance, the hydrogen evolution efficiency tends to be decreased. The high ERS flow over the electrode accelerates the reactant replenishment, reduces concentration polarization, and mitigates side reactions. However, the excessively high flow rates present several drawbacks. On the one hand, they require greater pumping power; on the other hand, they negatively affect the separation of hydrogen gas from the ERS in the gas–liquid separator, causing some fine hydrogen bubbles to remain entrained in the ERS [32]. The retained bubbles increase the ohmic resistance of the electrode compartment, adversely impacting the system performance. The optimizing flow rate thus requires balancing mass transfer efficiency with the pumping energy. While the higher flow rates improve mass transfer, they substantially increase pumping energy consumption [33], whereas lower flow rates may lead to concentration polarization and membrane fouling. It is believed that through proper flow rate optimization, mass transfer efficiency can be maintained while minimizing energy consumption as per the practical operating conditions.

### 3.3. Performance of the RED System Under the Comprehensive Optimum Conditions

**Figure 11:**
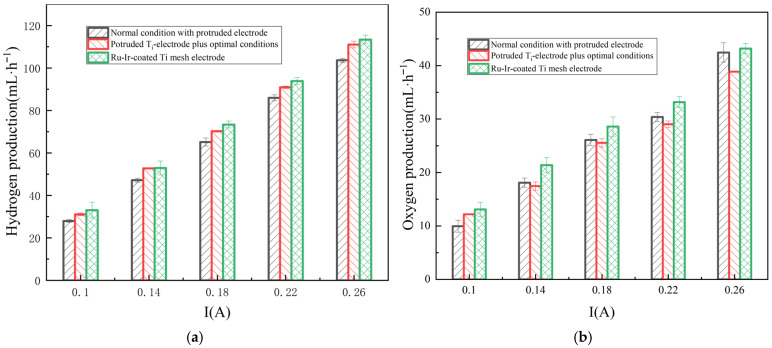
Variations in hydrogen (**a**) and oxygen (**b**) production with respect to the current in the hydrogen-electric cogeneration system using the mesh titanium-plate-coated oxide iridium and oxide ruthenium as electrodes and titanium redox electrodes under the optimized operating conditions.

The performance of the RED system under the comprehensive optimum conditions were experimentally investigated and are illustrated in Figure 11. The results confirm the feasibility of substituting the conventional Ru-Ir-coated Ti mesh electrodes with the non-noble Ti redox electrodes in RED hydrogen cogeneration systems. From an economic perspective, compare the prices of the new electrodes with those of the traditional precious metal electrodes on the market. Then, an analysis is conducted based on the proportion of metal prices in the market. By weighting each aspect equally at 50%, it is calculated that the new electrode can save 60% of the cost approximately. At a current of 0.22 A, the hydrogen evolution efficiency of the common Ti mesh electrode reached 78.56% of that achieved by the Ti-coated Ru-Ir electrode. In contrast, the RED system utilizing the specially structured Ti redox electrode attained a hydrogen production rate of 103.63 mL·min^−1^, which corresponds to 84.02% of the efficiency of the benchmark Ru-Ir electrode under the same operating condition. This outcome represents a fundamental possibility in the application of structured Ti redox electrodes for hydrogen production within RED systems.

Further, this study pursued enhanced hydrogen production in RED systems through the comprehensive optimization of the operational conditions. The experimental results reveal that certain operating parameters can improve hydrogen evolution efficiency, even at the cost of a slight suppression in output voltage and power density. Since maximizing hydrogen production was the primary goal, the marginal sacrifice in power density was considered justified. The comprehensive optimum operating condition was identified as an ERS temperature of 45 °C, a concentration of 0.1 M, and a flow rate of 50 mL·min^−1^. At 0.22 A, the RED system with the new electrode configuration that operated under the comprehensive optimum condition yielded a hydrogen evolution efficiency of 89.7% more than that using the previous Ru-Ir-coated Ti mesh electrode. This outcome underscores that through a combination of the structural design and operational conditioning, the performance of the less active Ti electrodes can be elevated to levels competitive with the noble metal Ru-Ir-coated Ti mesh electrode benchmarks. Consequently, for the future trajectory of RED-based hydrogen production—where industrial scalability and cost reduction are paramount—the adoption of such specially engineered, low-cost electrodes offers a practical and promising route to economically viable hydrogen generation.

## 4. Conclusions

This study presents a new spiked Ti redox electrode for application in RED hydrogen production systems and examines the effects of various operating conditions, including ERS temperature, concentration, and flow rate on system performance parameters such as output voltage, power density, hydrogen production, and oxygen production. In addition, this study combined the above-mentioned operating conditions and tested the performance that could be achieved by low-cost redox electrodes under such conditions.

- Based on the present study, the hydrogen evolution efficiency of conventional Ti mesh electrodes reaches only 78.56% of the Ru-Ir-coated Ti mesh electrode. The introduction of a spiked Ti mesh structure increases the efficiency by 5.46%.
- Building on the spiked structure of electrodes, increasing the ERS temperature from 15 °C to 45 °C enhances hydrogen production by 14.1%; raising the ERS concentration from 0.1 M to 0.7 M slightly increases oxygen evolution but reduces hydrogen production; decreasing the ERS flow rate from 130 mL·min^−1^ to 50 mL·min^−1^ cuts down the power generation performance but decreases the hydrogen evolution efficiency.
- Considering all operating parameters, the optimal conditions were determined as an ERS temperature of 45 °C, a concentration of 0.1 M, and a flow rate of 50 mL·min^−1^, under which hydrogen production reaches 89.7% of that of Ti-coated Ru-Ir electrodes. Moreover, the redox electrodes’ cost decreases over 60%, confirming the feasibility of replacing noble-metal electrodes with low-cost electrodes.

## Figures and Tables

**Table 1:** The main technical parameters of the measuring equipment used in the experiment.

Equipment	Model	Precision	Range	Manufacturer
Peristaltic pump for working solution	KCP	±5%	50~260 mL	Kamoer, Shanghai, China
Electrode liquid peristaltic pump	BT300-2J	0.1 rpm	0~300 rpm	Longer, London, UK
Conductivity meter	FiveEasy Plus	±0.5%	0~500 ms·cm^−1^	Mettler Toledo, Greifensee, Switzerland
Electrochemical workstation	CHI660E	0.2%	0~0.25 A	CH Instruments, Bee Cave, TX, USA
Current amplifier	CHI660C		±2 A	CH Instruments, USA
Electronic balance	AR124N	0.0001 g	0~120 g	Ohaus, Parsippany, NJ, USA
Constant temperature water tank	F34-ME	±0.05 °C	−30–150 °C	JULABO, Seelbach, Germany
Magnetic stirrer	RS-6DN	N/A	100~1500 rpm	ASONE, Osaka, Japan

**Table 2:** Specification parameters of wire mesh spacers.

Spacer Material	Thick Spacer δ	Porosity ε (%)	Shielding Coefficient	Resistance Coefficient
PET	220 μm	67	1.73	2.02

**Table 3:** Relevant performance parameters of ion-exchange membranes.

Membrane Type	δm (μm)	α * (%)	Ion-Exchange Capacity (meq·g^−1^)	AR ** (Ω·cm^2^)
CEM-Type 10	135	99	1.5	2
AEM-Type 10	125	95	1.8	1.7

** Membrane resistance is obtained by measuring a 0.5 M NaCl solution at 25 °C; * The selective permeability coefficient was measured at 25 °C through a KCl solution with a concentration range of 0.05–0.5 M.

## Data Availability

The original contributions presented in the study are included in the article. Further inquiries can be directed to the corresponding author.
